# The role of forest structure and composition in driving the distribution of bats in Mediterranean regions

**DOI:** 10.1038/s41598-022-07229-w

**Published:** 2022-02-25

**Authors:** Roberto Novella-Fernandez, Javier Juste, Carlos Ibañez, Jesús Nogueras, Patrick E. Osborne, Orly Razgour

**Affiliations:** 1 School of Biological Sciences, University of Southampton, Southampton, UK; 2 Terrestrial Ecology Research Group, Department for Life Science Systems, School of Life Sciences, Technical University of Munich, Freising, Germany; 3 Estación Biológica de Doñana (CSIC), Sevilla, Spain; 4 CIBER of Epidemiology and Public Health, CIBERESP, Madrid, Spain; 5 School of Geography and Environmental Science, University of Southampton, Southampton, UK; 6 University of Exeter, Exeter, UK

**Keywords:** Biogeography, Ecological modelling, Biogeography, Ecological modelling

## Abstract

Forests are key native habitats in temperate environments. While their structure and composition contribute to shaping local-scale community assembly, their role in driving larger-scale species distributions is understudied. We used detailed forest inventory data, an extensive dataset of occurrence records, and species distribution models integrated with a functional approach, to disentangle mechanistically how species-forest dependency processes drive the regional-scale distributions of nine forest specialist bats in a Mediterranean region in the south of Spain. The regional distribution patterns of forest bats were driven primarily by forest composition and structure rather than by climate. Bat roosting ecology was a key trait explaining the strength of the bat-forest dependency relationships. Tree roosting bats were strongly associated with mature and heterogeneous forest with large trees (diameters > 425 mm). Conversely, and contrary to what local-scale studies show, our results did not support that flight-related traits (wing loading and aspect ratio) drive species distributional patterns. Mediterranean forests are expected to be severely impacted by climate change. This study highlights the utility of disentangling species-environment relationships mechanistically and stresses the need to account for species-forest dependency relationships when assessing the vulnerability of forest specialists towards climate change.

## Introduction

Understanding the biogeographical processes that shape species diversity patterns is a principal aim of ecology with direct implications for predicting biodiversity responses to global environmental changes^1^. The operation of the several environmental filters that drive species distributions is dependent on spatial scale^2–4^. Climate is thought to drive broad-scale species patterns, while habitat preferences are thought to act generally at finer scales^5^, though under some circumstances they may have broader scale consequences^1^. Forests are key habitat types for biodiversity^6^. The spatial structure and composition of forests are known to contribute to shaping local-scale forest species assemblages (e.g.^7,8^), but the role of specific forest structure and composition characteristics in driving broader scale species distribution is less explored.

Mediterranean forests support high diversity and endemic species^9^. However, they are severely impacted by global change due to increased severity of summer droughts, heat stress and frequency of forest fires^10^, which can result in a range of consequences from structural and compositional changes to their replacement by scrubland^11,12^. Global change effects on forests are known to have major impacts on their associated fauna^13^, the extent of which is expected to depend on the strength of taxa-forest dependency relationships across spatial scales. A better understanding of the repercussions of these relationships beyond the local scale is therefore key for understanding future impacts of global change on the forest specialised fauna of Mediterranean regions.

Bats are the second most species rich order of mammals, with more than 1400 species^14^. The strong association between many bats and forests^15,16^ makes them an ideal model system to test broader-scale implications of forest characteristics on species distributions. Bat-forest dependency relationships are driven at least in part by species traits such as roosting preferences and flight-related morphology^16^. Most bats that roost in trees use crevices or cavities, formed through natural decay or excavated by woodpeckers, that provide suitable thermal conditions and protection from predators^17^. Trees with large diameter and height in early-mid stages of decay are generally preferred^18,19^, and broadleaf^20^ or softwood species that are prone to have cavities at earlier ages may provide more roosting opportunities. On the other hand, the forest structural types that forest bats, as aerial predators flying in a complex three dimensional space, can exploit is determined by bat morphology^21,22^. Wing aspect ratio affects turning speed, while wing loading affects flight speed, which indirectly affects manoeuvrability^23,24^. Only bats with low wing loading and low aspect ratio are able to fly at very low speeds, locate and capture prey in structurally cluttered forests^23,24^ with high tree densities, such as the young forests widespread in many Mediterranean areas. While bat-forest responses are usually studied at local spatial scales typically by comparing habitat use between different sites^21,25,26^, the broader-scale implications of these dependency relationships are still largely unknown^15,16,27^, despite recent increased research interest^28–30^.

We combine detailed fine-scale forest inventory data from the Mediterranean region of Andalusia (south of Spain) and a comprehensive dataset of occurrences of its forest-specialist bat fauna to (1) identify the importance of forest characteristics relative to climatic variables in determining regional-scale species’ distributions, and (2) determine whether those are driven by bat traits. We hypothesise that species traits will be key drivers of bat regional distributions. First, given that roosts are a crucial resource for bats^31^ and that tree-roosting species need a high number of suitable roosting trees due to frequent roost switching^17,32,33^, we predict that the regional distribution of tree roosting bats will be more strongly driven by the forest characteristics that promote the presence of tree roosts, compared to bats that roost in caves or human structures. Second, based on the ecomorphological relation between bat morphology and habitat use and the high prevalence of young forests with highly cluttered structure in the region, we also predict that among the bats that forage within forests, the regional distribution of species with morphological traits that do not enable them to fly in cluttered habitats (high wing loading and aspect ratio) will depend more strongly on forest characteristics related to forest spatial structure and clutter. We discuss the implications of the results for the conservation of forest specialist bats in Mediterranean regions.

## Methods

### Study system

**Figure 1:**
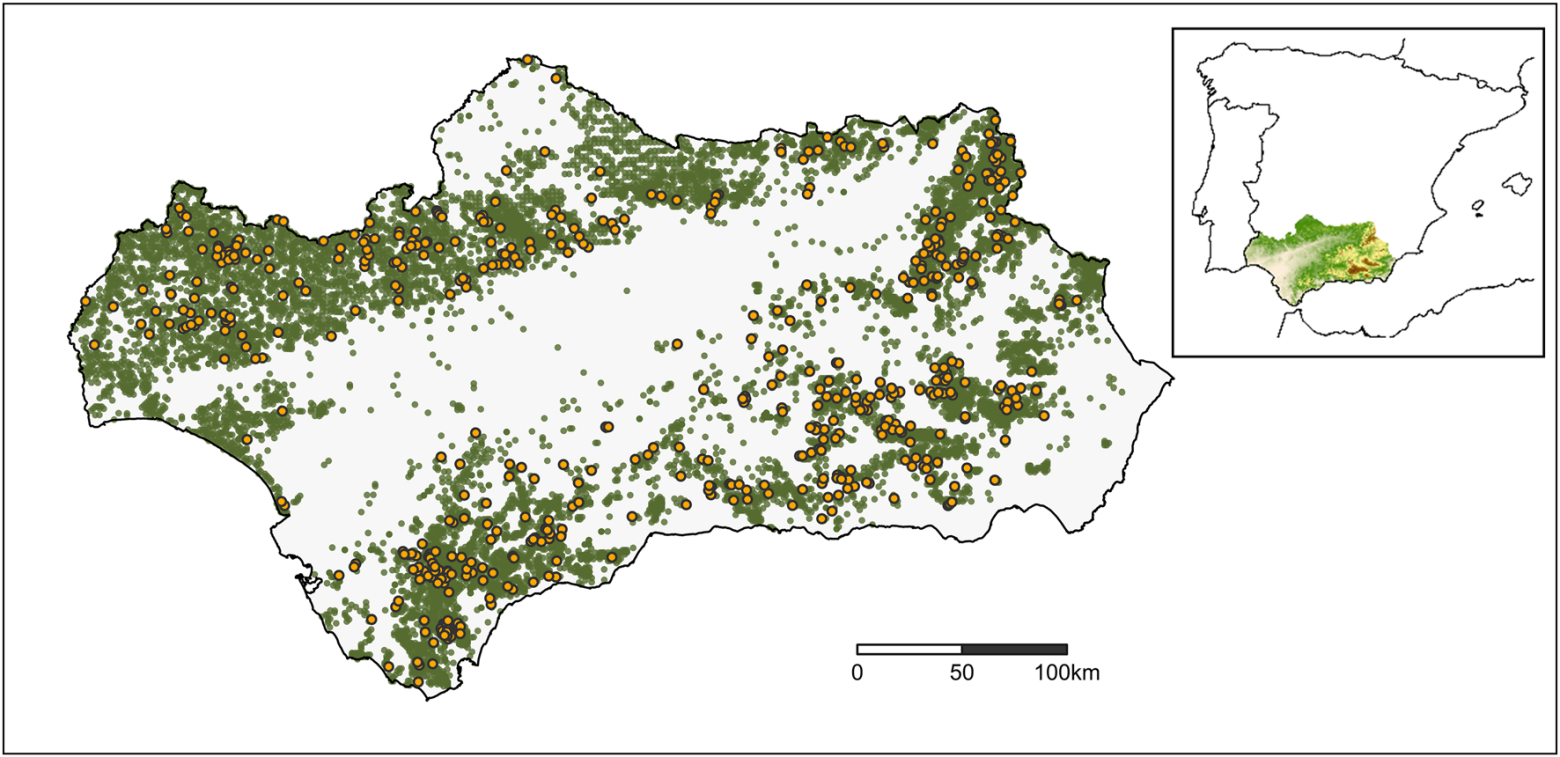
Study area (Andalusia, south of Spain) and occurrence records of the nine common forest bats in the region (orange dots) and forest plots (green dots). Areas without plots are not forested. Insert shows the location of Andalusia in the Iberian Peninsula and its elevation gradient from low elevations in green to high in brown (see Fig. S1). Generated using *ggplot2*^88^ within R 4.03 (www.r-project.org/).

The study area covers the 97,600 km^2^ of the Spanish region of Andalusia in the south of the Iberian Peninsula (Fig. 1). The region is climatically Mediterranean, though heterogeneous, with the mountain ranges of *Sierra Morena* in the north, and *Sierras Béticas* and *Penibética*s in the centre-east, and an elevation range extending from sea level to above 3000 masl (Fig. S1). The dominant forests in the region are mostly composed of the Mediterranean species *Quercus ilex*, *Pinus halepensis*, *Quercus suber*, and *Pinus pinea*, and in mountainous areas *Pinus pinaster*, *Pinus nigra*, and *Pinus sylvestris*. A large proportion of the forests are relatively young because they were exploited until the beginning of the second half of the twentieth century by the rural population, and many of the current forests were planted between 1940 and 1980^34^. We included in the study all forest bat species present in the region except *Myotis mystacinus* that is found in only two locations in the region^35^ (Table S1). *Myotis bechsteinii* and *Barbastella barbastellus* forage in forests and roost in tree holes and under loose bark, respectively^36^. *Myotis escalerai, Myotis emarginatus, Plecotus austriacus, Rhinolophus euryale,* and *Rhinolophus hipposideros* forage in forests but roost in caves and mines or anthropogenic structures^36,37^. Finally, *Nyctalus lasiopterus,* and *Nyctalus leisleri* roost in tree holes but represent an ecologically different group because they forage in open space above the forest canopy^36,38^. We used a comprehensive bat occurrence record database from EBD-CSIC (Spain) for the region of Andalusia, which was collected over several field seasons across the study area and offers a good representation of the spatial distribution of forest bats in the region. The spatial precision of the records was < 50 m. From the initial 15,680 records, we removed those sampled before 1987 to reduce the temporal difference with the forest sampling period in the region (1997–2007), resulting in 14,830 records retained. Records were obtained through a variety of sampling methods, mostly captures using mist-netting or harp-trapping in forests, caves, mine entrances, or buildings (Table S2). Only a few records (~ 1%) were based on acoustic identification due to the difficulties of acoustically distinguishing the three *Myotis* and two *Nyctalus* species, and recording the whispering bat, *P. austriacus*^39^. There are differences in the relative frequencies of sampling methods employed across species although the combination of sampling methods used together with the landscape-scale at which responses will be measured should provide a good overall representation of species´ habitat use. Some bats, particularly in continental and north Europe, show seasonal movements related to hibernation, which can entail habitat changes and may affect outputs of distribution models^40^. Most of our raw bat records (99%) corresponded to the long period of bat activity in the region (March-November). Moreover, none of the species is known to have regional migratory movements in our study system. For the four species with > 5% of observations belonging to the winter period of inactivity (hibernation) in the region, we validated that the distribution of their winter records fell within the summer ones.

We obtained information on the bat traits that most directly relate to the environmental filters considered from the literature (Table S3). Roosting ecology links directly to dependency on suitable trees for roosts, and wing loading (body mass divided by wing area) and aspect ratio (square of wingspan divided by area) are the most important descriptors of bat flight performance^23,24^. They are both negatively linked with manoeuvrability and are negatively related to habitat use in highly cluttered areas^23,24^ as shown in several local-scale community studies (e.g.^21,41^).

### Forest inventory data

We used the forest inventory database ‘*Tercer Inventario Nacional Forestal* (IFN3)’ (https://www.miteco.gob.es/es/biodiversidad/servicios/banco-datos-naturaleza/informacion-disponible/ifn3.aspx). The database contains forest data based on plots of 25 m of diameter distributed regularly over a 1 km grid covering the forested areas of Spain. Forest plots were sampled between 1997 and 2007, and each contains detailed variables describing structure and composition of forest. Trees in the database are defined as individuals of arborescent species with diameter at breast height (DBH) greater than 7.5 cm. We used R^42^ to process the database and obtain 52 plot-level forest structural and compositional variables that are potentially relevant for bats (Tables S4, S5). See Fig. 1 for location of forest plots in the working area.

We selected, from the full set of forest variables, a subset of 18 uncorrelated (r <|0.70|, Fig. S2, Dormann et al*.* 2013) quantitative forest variables and six categorical variables based on their contribution to single variable species distribution models and perceived importance for forest bats. We tentatively classified these forest variables into four mechanistic groups according to their link to bat ecology based on the literature (Table S6):

1. Roost availability: known to be driven by the size and age of trees, and the presence of dead trees^17^. We chose a DBH value of 425 mm as a threshold indicating large tree size given that only 6.5% of all the forest plots in the inventory database had a higher average value.
2. Structural heterogeneity: forest horizontal and vertical structural variability, high values are characteristic of mature forests^44^ and may provide higher resource richness^45^.
3. Forest clutter: the amount of spatial cluttering, driven by density of trees, canopy and understory cover. Higher cluttering restricts habitat use by bats depending on bat ecomorphology^23^.
4. Composition: the identity of tree and shrub species.

This classification is, however, not exclusive because some variables are inherently difficult to categorise. For instance, forests with older and larger trees usually are also structurally more heterogeneous.

### Data processing for species distribution models

#### Interpolation of forest inventory data

We used an interpolation process to characterise the forest area surrounding bat occurrence records based on data from the forest inventory plots (25 m in diameter separated by 1 km). For each bat occurrence record, we allocated a number of the closest forest inventory plots within a certain maximum distance and we summarised the values of all forest variables across them using the mean for quantitative or mode for categorical variables. The number of forest inventory plots allocated and their distance from the occurrence records varied depending on species’ home range sizes^36^. For the less mobile bat species, *M. bechsteini*, *M. emarginatus*, *M. escalerai*, *P. austriacus*, *R. eurale* and *R. hipposideros*, we selected the closest 2–5 forest plots within 3 km from the occurrence record. For *B. barbastellus,* we selected the closest 4–8 forest plots within 5 km, and for the more mobile *Nyctalus* species, we selected the closest 8–16 plots within 7 km. See Table S7 for interpolation parameters used and Table S8 for resulting interpolation descriptors.

To reduce spatial autocorrelation resulting from the duplication of forest data in nearby bat occurrences, prior to the forest data interpolation, we un-clustered bat occurrence records. The un-clustering distance depended on species´ home range sizes and previous interpolation parameters, from 500 m to 1000 and 2000 m (Table S7). The final dataset used in the models included 902 un-clustered records (Supplementary data 1, Figs. S3–S5).

The interpolation approach employed summarises forest characteristics within an area of several km^2^ around each occurrence record, and therefore it describes forest characteristics at the landscape-scale. This approach takes into account that bat occurrence records typically come from capture points within specific drinking, feeding, commuting or roosting locations within their home range. Note that this interpolation process cannot aim to fully describe the forest characteristics of bat home ranges because observations do not always correspond to the centre of the bat’s home range (which additionally, may vary depending on whether they correspond to a roost capture, foraging or drinking point). Additionally, bats often use certain core areas of their home range more intensively than others, and occasionally may also forage over larger distances than the ones used here. Despite of this, we believe that given the nature of our point-based data, this approach is the best option to capture an overall representation of the forested areas most immediately used by the bats. While interpolation parameters were selected based on species’ home ranges, we also aimed to maximise the density of background random points, which would be reduced if using larger interpolation distances given the relatively small study area. Processing of spatial point and polygon data was carried out using the R packages *sp*^46^ and *rgdal*^47^.

#### Interpolation of environmental data

In addition to the forest inventory data, we included in our models climatic and topographic variables. We selected from a subset of 15 climatic and topographic variables downloaded from WorldClim (www.worldclim.org) (Table S9) four uncorrelated variables (*r* <|0.70|) that summarise the effects of climate on bat species ranges (elevation, annual precipitation, maximum temperature of warmest month and temperature seasonality). We named this set of variables “climatic” for convenience (Table S6). All raster variables were downloaded at a resolution of 30 arc-seconds (~ 1 km). For consistency, we extracted the raster values of the four climatic variables to forest plot locations and followed the same interpolation process to the occurrence records as with the forest inventory data.

We also included a variable describing the proportion of forest as a measure of the importance of forest for bats irrespective of its structural and compositional characteristics. For that, we used the raster land cover map Corine Landcover 2000 (https://land.copernicus.eu/pan-european/corine-land-cover) at a resolution of 100 m. We reclassified all forest categories as 1 and the remaining land cover categories as 0. We created a layer of proportion of forest at a raster resolution of 2.5 km to roughly equal the spatial scale of the forest data after the interpolation process. We extracted values of proportion of forest cover directly to the occurrence records. All raster variable processing was done using the R package *raster*^48^.

#### Generating background point data

For each bat species we generated background data (pseudoabsences) by distributing an initial set of 10,000 random points across Andalusia, which we then thinned at 4 or 5 km (based on species’ home ranges, Table S7). The extent of this background area is adequate for SDMs considering that dispersal restrictions within the region are unlikely for flying animals like bats. We applied to these sets of un-clustered random background points the same process of forest data interpolation as for the presence occurrence records data. With this, we obtained for each bat species, the two datasets of environmental characteristics in presence locations and in pseudoabsence locations necessary to execute SDMs. We ensured that background data did not include presence locations by removing, from the forest plots available for background data, those closer than 4–5 km to the bat occurrence records (depending on species, Table S7). See Figs. S3–S5 for the location of occurrence and background data for each bat species; Table S8 for resulting interpolation descriptors to random points.

### Assessing bat responses to forest and climate

We generated SDMs to assess responses towards forest and climate using the Maxent algorithm (Phillips et al. 2006) in the R package *dismo*^50^. We chose this presence-only data framework because abundance data were not systematically collected in the study area, and therefore number of individuals recorded per location site is unlikely to represent population abundances. In Maxent, we used the sets of occurrence records with interpolated forest and climatic data as presences and the random points with interpolated forest and climatic data as pseudoabsences. Forest and climatic variables used are shown in Table S6.

We parameterised Maxent features and regularization values based on AICc scores (Table S8). For this, we used the R package ENMeval^51^ testing combinations of linear, quadratic and hinge features and regularization values between 1 and 3 to avoid model overfitting. We assessed model discrimination ability based on six cross-validations and the area under the receiver operator curve (AUC). To determine whether model discrimination ability was better than random, we compared for each bat species, model AUC test scores to null models. For this, we generated for each bat species, 100 different random datasets of presences with same number of records as the observed dataset, and followed with those the same downstream procedures of background data generation, interpolation and modelling as with observed data. Observed models with AUC test scores above 95% of confidence interval of null models’ AUC test scores were regarded as performing better than random^52^. We addressed our first objective by describing the contribution of the different mechanistic forest variable groups and single variables to each bat species’ model based on model training gain.

### Trait-based functional responses to forest

We tested whether functional traits of bats explained Maxent model training gain based on the hypotheses of our second objective (see^53–55^ for similar approaches). We first used linear models to test if roost ecology, wing loading and aspect ratio explained overall model training gain of species. We included in these models the number of occurrence records used as a covariate because it may affect Maxent model performance^56^. The first hypothesis of our second objective was that tree-roosting species have higher dependence on forests and towards characteristics that promote roost availability. Accordingly, we tested with an ANOVA if tree-roosting bat species showed higher summed training gain for all forest variables and separately for the mechanistic groups of forest variables and individual variables representing roost availability. Our second hypothesis was that, among the bats that exploit forests for foraging, the regional range of species with high wing loading and aspect ratio will be more restricted and therefore, dependant on specific forest characteristics, particularly those related to forest structural clutter. Accordingly, we tested whether there was a positive relationship between species wing loading and aspect ratio to the summed training gain of all forest variables and separately the individual and summed variables of the mechanistic group of cluttering. Note that the interpolated forest data used implies that bat-forest responses assessed in the models are measured at a landscape scale level, while the repercussions of these responses in species distribution are observed at a regional extent.

## Results

### Contribution of forest and climatic variables to species models

**Figure 2:**
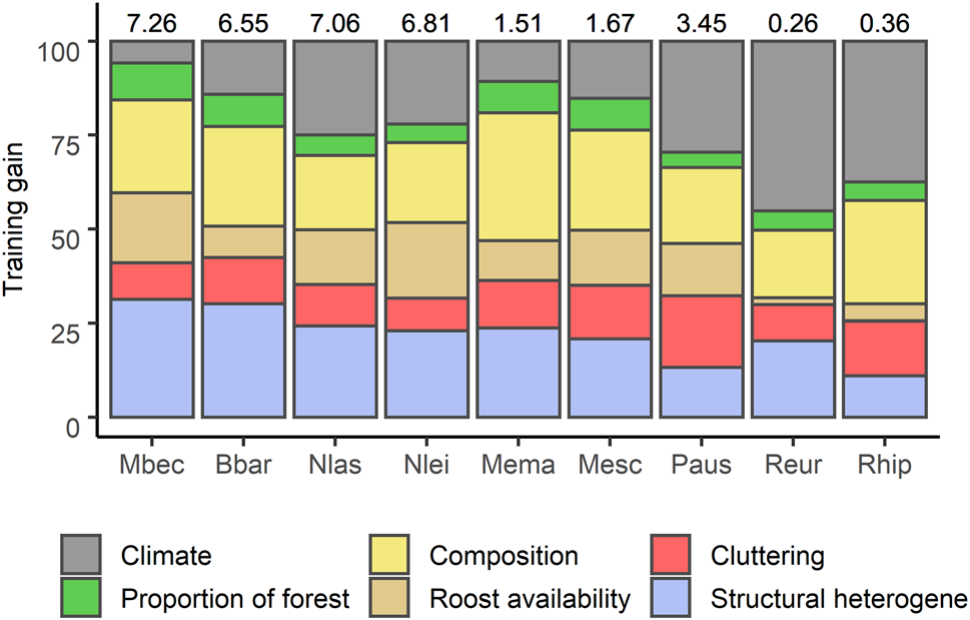
Relative training gain of the individual variables in the species distribution models for each bat species colour-coded according to mechanistic categories. Values on top show the total training gain of the model. Species abbreviations are shown in Table 1. Generated using *ggplot2*^88^ within R 4.03 (www.r-project.org/).

The discrimination ability of the SDMs varied between bat species. AUC_test_ scores were very high (> 0.85: *B. barbastellus*, *N. leisleri*, *M. bechsteini*, *N. lasiopterus*, *P. austriacus*) or high (0.70: *M. emarginatus*, *M. escalerai*) for most bat species, but considerably lower for *R. euryale* (0.63) and *R. hipposideros* (0.63; Table 1). However, in all cases AUC_test_ values were above 95% CI of null models (Fig. S6), and therefore performed better than random. Forest variables accounted for a greater proportion of model training gain (57.6–84.4%) than climatic variables (5.9–45.3%) in all bat species. Climate played a relatively greater role (> 25%) in the models of *R. euryale* (training gain = 0.12), *R. hipposideros* (0.13), *P. austriacus* (1.02), and *N. lasiopterus* (1.77)*.* Proportion of forest accounted for 4.1–9.8% of species´ training gain. The summed relative training gain of forest variables was highest (> 75%) for *M. bechsteinii* (6.12), *M. emarginatus* (1.22), *B. barbastellus* (5.06), and *M. escalerai* (1.27). Among the different mechanistic groups of forest variables, forest composition (17.9–34.0%) and structural heterogeneity variables (11.0 –31.2%) were the most important across bats*.* Forest cluttering variables contributed less (8.5–19.0%), yet slightly more than roost availability variables, which were more variable among species (1.9–20.2%), being most important (> 14%) for *N. leisleri*, and *M. bechsteinii* (Fig. 2)*.*

**Table 1:** Evaluation of the species distribution models generated for nine European forest bat species.

Abbreviation	Species name	Occurrence records	Training gain	Test AUC	95% CI AUC_Test_ null models
Bbar	*Barbastella barbastellus*	16	1.92	0.975	0.25–0.70
Mbec	*Myotis bechesteinii*	49	1.68	0.922	0.40–0.66
Mema	*Myotis emarginatus*	98	0.52	0.749	0.40–0.61
Mes	*Myotis escalerai*	115	0.49	0.710	0.42–0.58
Nlas	*Nyctalus lasiopterus*	48	1.47	0.913	0.33–0.63
Nleis	*Nyctalus leisleri*	48	1.48	0.938	0.39–0.67
Paus	*Plecotus austriacus*	123	1.00	0.855	0.44–0.61
Reur	*Rhinolophus euryale*	173	0.18	0.629	0.45–0.58
Rhip	*Rhinolophus hipposideros*	322	0.16	0.626	0.47–0.58

Number of occurrence records used, overall training gain and test AUC values for each bat species compared with test AUC of null models (confidence interval 0.05–0.95). Species abbreviations shown.

Identity of the dominant tree species was the most important composition variable and one of the most important variables in the models (Fig. S7). *Quercus pyrenaica* was the dominant tree species most strongly preferred by four bat species (*M. bechsteinii*, *M. emarginatus, M. escalerai*, and *P. austriacus*). *B. barbastellus* and *M. escalerai* also responded strongly towards *Pinus nigra.* Conversely, *N. lasiopterus*, N. *leiserli*, *R. euryale* and *R. hipposideros* did not show clear preferences towards dominant tree species. The least preferred forest types for most bat species were those composed of the Mediterranean species *Pinus halepensis*, *Pinus pinea* and *Quercus ilex* (Fig. S8). Tree richness was also a very important composition variable, with the probability of occurrence of most bat species increasing at higher tree richness values (Fig. S7). Among the variables of forest structural heterogeneity that contributed more to the models are included the range of diameter at breast height (DBH) and tree height (Fig. S7). The former was very important for the four tree roosting species (*M. bechsteinii*, *B. barbastellus*, *N. lasiopterus*, *N. leisleri*), whose probability of occurrence increased at higher tree diameter sizes (Fig. S10). Tree height range was also very important for *M. bechsteinii* and *B. barbastellus* (Fig. S7), and most species also responded positively (Fig. S11). Responses towards percent of plantations varied among species, from negative and neutral in most bats to positive in *M. emarginatus* and *P. austriacus* (Fig. S12). Tree cover was the most important forest cluttering variable (Fig. S7), and responses were variable across species (Fig. S13). The most important roost availability variable were density of trees with DBH > 425 mm, which was more important for the tree roosting species with the exception of the bark roosting *B. barbastellus* (Fig. S7), and density of soft wood trees with DBH > 425 mm, which was important for *M. bechsteinii* and *B. barbastellus* (Fig. S7). Density of dead trees had very low training gain in all species’ models (Fig. S7). See Fig. S14 for variable importance based on AUC.

### Trait-based functional responses to forest structure and composition

#### Role of bat roosting ecology in driving bat-forest relationships

**Figure 3:**
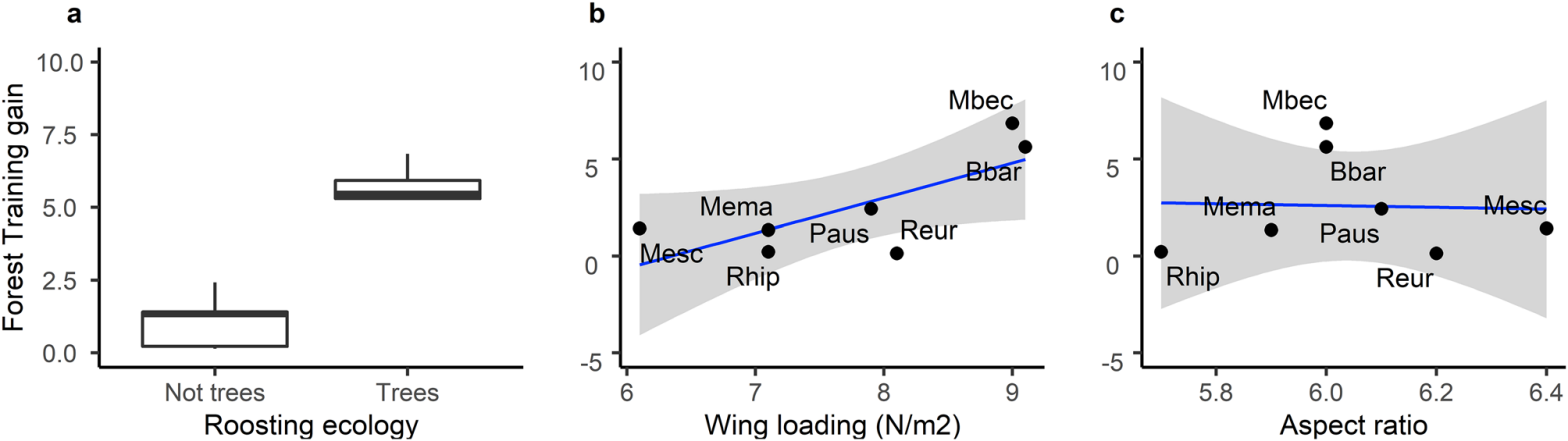
Relation between summed training gain of forest variales in the species distribution models depending on species functional traits (roosting ecology (**a**), wing loading (**b**) and aspect ratio (**c**)). Generated using *ggplot2*^88^ within R 4.03 (www.r-project.org/). The two *Nyctalus* species are excluded from plots (**b**, **c**) because the ecomorphological relation between bat morphology and habitat structure is only relevant for species that forage within the forest and these two species are open space foragers that forage above the forest canopy.

Bat roosting ecology explained the differences in the training gain of models across species. Tree-roosting bats had higher summed training gain than non-tree roosting bats (Linear Model: *F*_2,6_ = 42.46, *p* < 0.001, *R*^2^ = 0.91, Roosting ecology: *p* = 0.002), and differences were not affected by the number of occurrence records (*p* = 0.159). Tree-roosting bats had larger contribution of proportion of forest (ANOVA: F_1,7_ = 24.41, *R*^2^ = 0.74, *p* = 0.002), and summed contribution of forest variables in their models (ANOVA: *F*_1,7_ = 64.65, *R*^2^ = 0.89 *p* < 0.001; Fig. 3a), including contribution of all four forest mechanistic groups of variables separately: roost availability variables (ANOVA: *F*_1,7_ = 20.58, *R*^2^ = 0.71, *p* < 0.001), composition (ANOVA: *F*_1,7_ = 54.72, *R*^2^ = 0.87, *p* < 0.001), heterogeneity (ANOVA: *F*_1,7_ = 95.77, *R*^2^ = 0.92, *p* < 0.001), and cluttering variables (ANOVA: *F*_1,7_ = 12.77, *R*^2^ = 0.59, *p* = 0.009). When looking at the individual roost availability variables, the training gain of density of trees with DBH > 425 mm was higher for tree-roosting bats than for non tree-roosting bats after multiple comparison holm correction (ANOVA: *F*_1,7_ = 15.07, *p adjusted* = 0.040, Table 2, Fig. S15).

**Table 2:** Variable importance of the roost availbility variables for tree roosting versus non-tree roosting bats.

Forest variable	Non-tree roosting Mean ± SD	Tree roosting Mean ± SD	*F*_1,7_	*p* adj
Tree DBH	0.009 ± 0.007	0.060 ± 0.068	2.92	0.394
Tree h	0.009 ± 0.012	0.080 ± 0.070	5.05	0.250
Dead den	0.001 ± 0.001	0.001 ± 0.002	0.61	0.918
> 425 DBH den	0.038 ± 0.036	0.522 ± 0.281	15.07	0.042*
SoftW > 425 den	0.075 ± 0.074	0.272 ± 0.169	5.59	0.250
Dev stage	0.001 ± 0.002	0.124 ± 0.097	8.30	0.142
Wood density	0.048 ± 0.090	0.017 ± 0.012	0.47	0.918

Mean training gain and standard deviation (SD) in the species distribution models of each roosting group, ANOVA test results (F value and adjusted p value after Holm correction for multiple comparisons). *Notes detectable effect at *p* < 0.05. (Tree DBH: average diameter at breast height of trees in the plot, Tree height: average height of trees, Dead den: Density of dead trees, > 425 DBH den: density of trees with diameter at breast height larger than 425 mm, SoftW > 425 den: density of trees of soft wood larger than 425 mm, Dev stage: development phase of the main tree species, Wood density: weighted average wood density of trees; Table S5).

#### Role of bat flight morphology in driving bat-forest relationships

Tree roosting bats had higher wing loading values (Table S3), and therefore, this variable was not independent from roosting ecology. Wing loading of forest-foraging bats narrowly did not explain species’ model training gain, which was also unaffected by the number of records used (Linear Model: *F*_2,4_ = 6.73, *p* = 0.052, Wing loading: *p* = 0.144, number occurrence records: *p* = 0.132). Species wing loading did not explain the training gain of the variable proportion of forest in the models (*F*_1,5_ = 5.53, *p* = 0.066) nor the summed training gain of all forest variables, although there was a marginal trend (F_1,5_ = 6.20, *p* = 0.055; Fig. 3b). When considering separately the mechanistic groups of forest variables, wing loading was only positively correlated with training gain of structural heterogeneity variables (F_1,4,_ = 7.00, R^2^ = 0.50, *p* = 0.045). Aspect ratio did not explain species’ model training (F_2,4,_ = 4.80, *p* = 0.086), training gain of proportion of forest (*F*_1,5_ = 0.01, *p* = 0.916), summed training gain of forest variables (*F*_1,5_ = 0.01, *p* = 0.936; Fig. 3c) nor any mechanistic group of forest variables (*p* > 0.05). Contrary to predicitons, neither wing loading nor aspect ratio were related to the contribution of any specific cluttering variable (*p* > 0.05).

## Discussion

By integrating detailed forest inventory data with species distribution models and a functional approach, we disentangle the regional-scale implications of bat-forest dependency relationships. We show that forest structure and composition play primary roles in driving the distribution of forest-specialist bats in Mediterranean regions, and that roosting ecology drives the strength of these bat-forest responses. In contrast, despite the known local-scale effects of wing loading and aspect ratio on bat´s forest use^57^, we found no conclusive evidence for their role in driving regional-scale bat distributions. The relationship found between the regional distribution of forest bats and forest characteristics suggests that future changes in forest structure driven by climate change may impact such forest specialist fauna at broad regional scales.

### Regional-scale implications of bat-forest associations

Forest characteristics are known to play important roles in driving the community assemblage patterns of various taxonomic groups^58–60^. Yet only few studies have addressed the role of these in shaping regional-scale species distributions, for example, forest maturity was shown to drive range expansions in forest birds^61,62^. Our results show that forest variables play a primary role in shaping the regional distribution of forest bats in Andalusia. The strong relative importance of forest characteristics seen in this study occurs despite the high abiotic heterogeneity of the region, which has a very broad elevation range (0–3000 masl) and different climates. Responses towards forest characteristics were strong drivers of bat species distributions despite other habitat features not considered within the scope of this study, such as the wider landscape structure are known to be relevant for bats^27,28^. The contribution of forest features to species distribution, however, varied across bat species. It was high for *M. bechsteinii*, *M. emarginatus*, *B. barbastellus*, and *M. escalerai,* while lower for *P. austriacus, N. lasiopterus, N. leiseri* and particularly *R. euryale* and *R. hipposideros.* The models of the latter two species did not perform as well as the others, suggesting that they are generalists in the region. This is in line with previous studies showing that *R. euryale* and *R. hipposideros* forage in a variety of habitats, including hedgerows and forest edges^63,64^. Even though different proportion of capture methods were used across species (i.e. cave roosting bats were sampled in roosts in higher proportion than tree-roosting species) it is unlikely that this may cause relevant biases in the responses observed because the landscape scale used to measure bat responses summarises forest use at broader scale than the immediate surroundings of the roost.

Identity of dominant tree species was ones of the most important forest variables affecting the distribution of forest bats in our study. This variable was previously identified as important for driving patterns of richness and abundance of bird communities in boreal forests^58^. *P. nigra* and *Q. pyrenaica* stood out as preferred tree species for most bats, even though the second is uncommon in the region. The link between forest fauna and certain tree species may, however, be related to a wider set of forest characteristics, from associated forest structures^65^ to differences in arthropod prey composition and abundances^66,67^. Furthermore, it may be difficult to tease apart bat responses toward specific tree species from responses to climate due to the associations between tree species and specific climatic conditions^68^. The observed general under-selection of forests dominated by the Mediterranean tree species *Q. ilex,* and *P. halepensis* had been reported previously^20^, and may be due to the generally poorly developed structures of Mediterranean forests due to their slow growth rates and high perturbation regime due to anthropogenic disturbance^69^. Probability of occurrence of bat species increased with tree species richness, a variable commonly reported as driving richness patterns and abundances of other taxa, such as birds^70^ or herbivorous arthropods^71,72^. This relationship may be interpreted as result of increased diversity of trophic resource availability across trophic levels^71,72^, and for tree roosting bats it may also represent increased roost availability through the presence of, usually less dominant, softwood tree species^73^.

Forests with a mature structure are thought to promote biodiversity^58,74^ and support higher bat richness and abundance^16^. Accordingly, in our study, variables describing structural heterogeneity that are characteristic of mature forests were particularly important for most bat species. Forest structural heterogeneity could be linked to higher diversity of microhabitats that provide more resources^45^. The presence of trees of greater diameter and height and well developed forest structures were also important in the models as it has been shown in other studies^74^, though less than structural heterogeneity variables. The responses towards tree density were negative for almost all bat species, as expected based on ecomorphological constraints. The presence of dead trees is regarded as one of the key forest characteristics that provides suitable roosting habitats for tree roosting bats^17^, and therefore the landscape-level density of standing dead trees can have a positive impact on bat abundance^75^. However, in this study density of dead trees was not identified as an important variable. This might be because the forest inventory database did not specify whether dead trees were standing or lying on the ground, the latter not being a valid roosting resource for temperate forest bats. Finally, tree plantations are widespread in European forest landscapes, and therefore their contribution to biodiversity has important ecological repercussions. We found overall slightly negative associations of bats with plantation forests, though not a complete avoidance, and even positive responses by some of the more generalist species, *M. emarginatus* and *P. austriacus,* that matches previous studies^76,77^.

### Role of species traits in driving bat responses towards forests

Bat roosting ecology explained the strength of bat responses towards forest, and therefore the distribution area of tree roosting bats was more strongly driven by forest characteristics compared to that of bats that do not roost in trees. Tree roosting bats showed stronger dependency on proportion of forest, overall forest variables and all mechanistic groups of forest variables: forest composition, structural heterogeneity, clutter, and in line with our first prediction, roost availability variables. Among those, the single variable density of trees with DBH > 425 mm, had a stronger effect on the regional distribution of tree-roosting bats compared to bats that roost in caves and human structures. This variable relates to an advanced stage of forest structure development, which for example, has been identified as an important driver of habitat preferences of breeding birds in temperate forests in Germany^74^.

The role of roost availability in limiting the distributions of tree-roosting bat that we report is not surprising when considering the high number of roosts that these require due to frequent roost switching^17,32,33^ alongside the scarcity of mature forests in Mediterranean regions. Mediterranean forest may provide less of some of the key structural variables linked to roost availability compared to temperate forests, and this may explain the scarcity of tree-dwelling species in the south of the Iberian Peninsula^78^. The strength of forest characteristics in driving the distribution of tree roosting bats that we found is likely generalisable to other Mediterranean regions which contain forests with similarly characteristics, and may be a driver of continental-level biogeographical patterns of forest-bats across Europe and other Mediterranean ecoregions. To better understand such biogeographical patterns, there is a need for more studies that combine fine-scale forest structure data, such as forest inventory data, and LIDAR data^57^ over large spatial extents.

Bats with wing morphologies characterised by high aspect ratio and wing loading have limited access to spatially cluttered areas due to reduced manoeuvrability^23,24^. Several local-scale studies show how this drives their habitat use^21,41,57^. In our study system, we expected to find a positive relationship between wing loading and dependency on forest variables, particularly those describing forest clutter, because higher wing loading would restrict bats distributions to forested areas with suitable amount of clutter. We found a weak relationship between wing loading of forest foraging bats and their dependence on forest variables, and only a positive relationship between bat wing loading and training structural heterogeneity variables, though not with clutter as would be expected if, as predicted, the regional distribution of species with higher wing loading was limited by forest structure. The relationship was, moreover, not observed with aspect ratio and neither variable explained the importance of any of the individual variables related to cluttering. Therefore, our data does not provide a clear support for this hypothesis, and we interpret that the weak relationship between wing loading and forest variables is most likely an artefact caused by the strong correlation between bat wing loading and roosting ecology. This may indicate that bat-forest ecomorphological relationships primarily contribute to shaping only local-scale habitat use patterns (bellow landscape-scale), as observed in local scale studies, and those patterns disappear at slightly larger landscape-scales. This has been previously observed in communities of tropical forest bats in West Africa, where their functional structure based on habitat use and foraging guild was driven by vegetation structure at the local-scale, but was random at the landscape-scale^27^.

It is worth mention that in addition to the roost and flight ecomorphological mechanisms considered in this study, bats are expected to respond to forests based on other mechanisms, such as trophic resource availability. For instance, prey abundance can drive bat activity after forest structure filters bat species depending on their flight-related traits^79^. While trophic-habitat relationships are not often addressed (e.g.^22,80^), considering them will be important for a future better understanding on bat-forest relationships.

### Conclusion and implications for conservation

While mature forest characteristics are known to increase the quality of forest habitats for bats^16^, we show that the repercussions of such positive relationships, particularly among forest bats that roost in trees, are far beyond local scales and can be the main drivers of at least medium scale biogeographic patterns of species’ distributions. This further stresses the importance that forest structure has for the conservation of its associated fauna. Mediterranean forests are, with their slow growth rates together with high perturbation regime, often unlikely to reach the mature stages that are highly valuable for tree-roosting bats. These forest are, moreover, highly vulnerable to climate change^12^ and are expected to be severally impacted via increased drought, pathogens and wildfires^81,82^, which may result in their replacement by more drought-adapted scrublands^11^. While future range-shift predictions of bats are often solely based on climate (e.g.^83,84^), the strong dependency of forest bats on forest structure that we show suggests that neglecting to account for the interplay between climate effects and bat habitats may produce biased predicted impacts, which could be stronger when considering forest changes. This effect has been seen in birds and mammals worldwide, where the interaction between climate and landcover changes is estimated to produce stronger synergic impacts^85^. This study therefore highlights the need for better consideration of species-forest dependency relationships when assessing climate change vulnerability of forest specialists. Forest management policies that aim to promote mature forest structures that foster quality habitats for biodiversity while increasing forest resilience towards climate change^86,87^ can be a key tool to promote broader-scale conservation of Mediterranean forests and their associated fauna.

## Supplementary Information

Supplementary Information 1.

Supplementary Information 2.

## Data Availability

Bat location records are available as Supplementary data 1. Forest and environmental data are publicly available to download (https://www.miteco.gob.es/es/biodiversidad/servicios/banco-datos-naturaleza/informacion-disponible/ifn3.aspx).
