# Supplementary material for: The role of forest structure and composition in driving the distribution of bats in Mediterranean regions
Source: Sci Rep. 2022 Feb 25;12:3224. doi: 10.1038/s41598-022-07229-w (PMC8881505; doi:10.1038/s41598-022-07229-w)
Supplement: Supplementary file 1 — Supplementary Information 1. [file 41598_2022_7229_MOESM1_ESM.pdf]

Roberto Novella-Fernandez[1,2*], Javier Juste[3,4], Carlos Ibañez[3], Jesús Nogueras[3], Patrick E. Osborne[5], Orly Razgour[1, 6]

Supporting information for: The role of forest structure and composition in driving the distribution of bats in Mediterranean regions

1-School of Biological Sciences, University of Southampton, Southampton, UK

2- Technical University of Munich, Terrestrial Ecology Research Group, Department for Life Science Systems, School of Life Sciences, Freising, Germany

3- Estación Biológica de Doñana (CSIC), Sevilla, Spain.

4- CIBER of Epidemiology and Public Health. CIBERESP, Spain.

5- School of Geography and Environmental Science, University of Southampton, Southampton, UK

6- Biosciences, University of Exeter, Exeter, UK

*r.novella@tum.de, ORCID ID: 0000-0003-4013-0646

Table S1 – Bat species included in the study and their dependency on forests. *excluded due to extremely restricted presence in the area.

		Foraging habitat dependency		
		**Limited**	**Facultative**	**Exclusive**
**Roost in tree dependency**	**Limited**		*R. euryale*<br>*P. austriacus*	*R. hipposideros*<br>*M. escalerai*
	**Facultative**			*M. emarginatus*<br>*M. mystacinus**
	**Exclusive**	*N. leisleri*<br>*N. lasiopterus*		*B. barbastellus*<br>*M. bechsteinii*

Table S2- Origin and number of occurrence records used for each bat species. Abbreviations shown in Table 1.

	Bbar	Mbec	Mema	Mesc	Nlas	Nlei	Paus	Reur	Rhip
**Cave-Mine: acoustic-visual**	0	3	176	178	0	0	73	950	881
**Cave-Mine: Capture**	18	12	225	492	0	2	165	767	78
**Colection**	2	2	3	4	6	13	15	2	4
**Field: Wind generators**	0	0	1	0	41	17	0	0	0
**Field: acoustic-visual**	7	2	0	0	81	22	2	4	11
**Field: capture**	99	308	73	236	1145	906	441	3	18
**Human construction: acoustic-visual**	0	0	37	1	0	0	10	34	175
**Human construction: Capture**	1	6	62	66	2	4	22	64	1805
**Literature**	1	0	2	5	0	1	31	16	27

**Batbox on tree**	2	29	0	3	4063	6	0	0	0
**Batbox Traking**	0	0	0	0	867	0	0	0	0

Table S3 – Functional traits of the bat species included. Aspect ratio describes the square of the wingspan divided by the wing area. Wing loading describes the weight of the animal divided by the wing area.

**Bat Species**	**Roost ecology**	**Aspect ratio**	**Wing loading (N/m^2)**	**Source**
M. bechsteinii	Trees	6	9	(Norberg & Rayner 1987)
B. barbastellus	Trees	6	9.1	(Norberg & Rayner 1987)
N. lasiopterus	Trees	7.2	14.6	(Ibáñez et al. 2001)
N. leisleri	Trees	7.9	19.3	(Norberg & Rayner 1987)
M. emarginatus	Caves-buildings	5.9	7.1	(Norberg & Rayner 1987)
M. escalerai	Caves-buildings	6.4	6.1	(Norberg & Rayner 1987)
P. austriacus	Caves-buildings	6.1	7.9	(Norberg & Rayner 1987)
R. hipposideros	Caves-buildings	5.7	7.1	(Norberg & Rayner 1987)

Supplementary tables and figures

Table S4 – Forest variables thought to be relevant for bat ecology that have been generated from the Forest Inventory database. For each variable, the following information is presented: average, minimum, and maximum values, type of variable, mechanistic group and description.

	Name	Units	Mean	Min	Max	Type	Calculated?	Mechanism	Description
1	Forest type					Cat	As came	Heterogeneity	Morphostructural type.
2	Spatial distr					Cat	As came	Cluttering	Spatial distribution.
3	Sp comp					Cat	As came	Composition	Type of specific composition.
4	Total C	(%)	88.9	2	100	Quant	As came	Cluttering	Total vegetation cover, estimated in percent.
5	Tree C	(%)	53.5	0	100	Quant	As came	Cluttering	Tree cover, estimated in.
6	Tree sp					Cat	Calculated	Composition	Identity of main species. Grouping rare species (dominant <1.5 % plots) in others.
7	Dev stage					Cat	As came	Roost	Development phase of the main tree species.
8	Sp1 DBH struct					Cat	As came	Heterogeneity	Structural heterogeneity category (regular to irregular) of the main tree species.
9	Sp1 occupancy		7.6	1	10	Quant	As came	Composition	Percentage of occupation of the main tree species.
10	coniferous den	(trees/ha)	246.1	0	4877.21	Quant	Calculated	Cluttering	Density of trees of coniferous species.
11	Trees/ha	(trees/ha)	516.8	0	8994.02	Quant	Calculated	Cluttering	Tree density (>75 DBH) in the plot.
12	% coniferous	(prop)	0.5	0	1	Quant	Calculated	Composition	Proportion of coniferous trees
13	Sp1 density	(trees/ha)	449.7	5.09	8994.02	Quant	Calculated	Cluttering	Density of the species with more tree density.
14	Sp1 D	(%)	0.9	0.2	1	Quant	Calculated	Composition	Dominance of tree Sp1 (rel. tree density).
15	Broadleaved	(trees/ha)	261.6	0	8994.02	Quant	Calculated	Composition	Density of broadleaved tree species.
16	Tree DBH	(mm)	217.4	75	3072.8	Quant	Calculated	Roost	Average DBH of trees in the plot.
17	% broadleaved	(prop)	0.5	0	1	Quant	Calculated	Composition	Proportion of broadleaved trees.
18	Min tree DBH	(mm)	151.8	25	3071	Quant	Calculated	Roost	Diameter of the thinner tree.
19	Max tree DBH	(mm)	401.1	75	3071	Quant	Calculated	Roost	Diameter of the largest tree.
20	Tree DBH ra	(mm)	249.3	0	2524	Quant	Calculated	Heterogeneity	DBH range. Thicker - thinner tree.
21	DBH categories	1 to 7	3.1	0	7	Quant	Calculated	Heterogeneity	Number of diametrical classes.
23	% < 225	(%)	64.1	0	100	Quant	Calculated	Roost	Relative density of trees (%) with DBH less than 225 mm.
24	% < 325	(%)	82.4	0	100	Quant	Calculated	Roost	Relative density of trees (%) with DBH less than 325 mm.
25	% > 425	(%)	8.6	0	100.1	Quant	Calculated	Roost	Relative density of trees (%) with DBH larger than 425 mm.
26	% > 525	(%)	4.0	0	100.1	Quant	Calculated	Roost	Relative density of trees (%) with DBH larger than 525 mm.
27	% > 625	(%)	1.9	0	100.1	Quant	Calculated	Roost	Relative density of trees (%) with DBH larger than 625 mm.
28	>425 DBH den	(trees/ha)	421.2	0	8753.5	Quant	Calculated	Roost	Density of trees with DBH less than 225 mm.
29	>425 DBH den	(trees/ha)	485.4	0	8994	Quant	Calculated	Roost	Density of trees with DBH less than 325 mm.

	Name	Units	Mean	Min	Max	Type	Calculated?	Mechanism	Description
**30**	>425 DBH	(trees/ha)	9.7	0	285.2	Quant	Calculated	Roost	Density of trees with DBH larger than 425 mm.
**31**	>425 DBH den	(trees/ha)	3.5	0	178.3	Quant	Calculated	Roost	Density of trees with DBH larger than 525 mm.
**32**	>425 DBH den	(trees/ha)	1.4	0	122.2	Quant	Calculated	Roost	Density of trees with DBH larger than 625 mm.
**33**	Tree H	(m)	9.2	1.5	41.4	Quant	Calculated	Roost	Average height of trees.
**34**	Min tree height	(m)	6.6	1.4	39.5	Quant	Calculated	Roost	Height of the smallest tree.
**35**	Max tree height	(m)	13.4	1.5	59	Quant	Calculated	Roost	Height of the largest tree.
**36**	Tree H ra	(m)	6.8	0	51	Quant	Calculated	Heterogeneity	Tree height range. Highest - shortest tree.
**37**	Dead den	(trees/ha)	16.4	0	4138	Quant	Calculated	Roost	Density of dead trees (Not specify if ground or standing).
**38**	% dead trees	(trees/ha)	2.9	0	100.1	Quant	Calculated	Roost	Relative density of dead trees.
**39**	Tree R		4.4	1	24	Quant	Calculated	Composition	Tree species richness in the plot of 25m.
**40**	Underst C	(%)	54.8	0	347	Quant	Calculated	Cluttering	Understory cover, estimated as the sum of % cover of each scrub species.
**41**	Underst R		4.6	1	26	Quant	Calculated	Composition	Richness of shrub species.
**42**	Underst H	(m)	0.9	0	10.35	Quant	Calculated	Cluttering	Average height of shrub.
**43**	Underst max height	(m)	1.3	0	20	Quant	Calculated	Cluttering	Height of the highest shrub species.
**45**	Small tree density	(trees/ha)	732.4	0	17189	Quant	Calculated	Cluttering	Density of small trees (<7.5 DBH) on a 5 m plot.
**46**	Small tree sp1 den	(trees/ha)	951.5	127	15661	Quant	Calculated	Cluttering	Density of the main species of lesser trees.
**47**	Small tree height		34.7	2	170	Quant	Calculated	Cluttering	#N/D
**48**	Regeneration richness		2.6	0	16	Quant	Calculated	Composition	Species richness of regeneration.
**49**	Wood density	(g/cm3)	0.7	0.43	0.991	Quant	Calculated	Roost	Weighted average wood density of trees.
**50**	Wood density >425	(g/cm3)	0.7	0.43	0.99	Quant	Calculated	Roost	Weighted average wood density of trees larger than 425 mm (Table S4).
**51**	SoftW tree den	(trees/ha)	288.7	0	4877.21	Quant	Calculated	Roost	Density of trees of soft wood (Table S4).
**52**	HardW tree den	(trees/ha)	196.0	0	5124.79	Quant	Calculated	Roost	Density of trees of hard wood (Table S4).
**53**	SoftW tree prop	(%)	0.6	0	1	Quant	Calculated	Roost	Relative density of trees of soft wood (Table S4).
**54**	SoftW >425	(trees/ha)	5.6	0	285.21	Quant	Calculated	Roost	Density of trees of soft wood (Table S4) larger than 425 mm.

Table S5 – Wood density data for the tree species in the INF3 database. Used to calculate variables on wood density of trees (Softwood tree density, Harwood tree density, Softwood tree prop, Softwood >425).

Species	Wood density (g/cm3)	Category	Source
Populus nigra	0.43	Soft	1
Tilia cordata	0.44	Soft	1
Populus x canadiensis	0.457	Soft	2
Abies alba	0.51	Soft	1
Alnus glutinosa	0.51	Soft	1
Pinus radiata	0.515	Soft	3
Pinus uncinata	0.52	Soft	1
Pinus pinaster	0.52	Soft	1
Populus alba	0.53	Soft	2
Pinus sylvestris	0.55	Soft	1
Populus tremula	0.56	Soft	1
Eucalyptus globulus	0.57	Soft	4
Pinus pinea	0.59	Soft	1
Castanea sativa	0.59	Soft	1
Betula pendula	0.6	Soft	1
Pinus halepensis	0.61	Soft	1
Pinus nigra	0.62	Soft	1
Juniperus thurifera	0.648	Soft	5
Platanus hybrida	0.66	Soft	1
Quercus robur	0.675	Hard	3
Fraxinus excelsior	0.68	Hard	1
Fagus sylvatica	0.69	Hard	1
Fraxinus angustifolia	0.69	Hard	2
Acer campestre	0.69	Hard	3
Quercus petraea	0.73	Hard	1
Acer opalus	0.73	Hard	1
Quercus canariensis	0.76	Hard	1
Quercus pubescens (Q. humilis)	0.77	Hard	1
Robinia pseudoacacia	0.77	Hard	3
Quercus cerrioides	0.78	Hard	1
Quercus faginea	0.79	Hard	1
Arbutus unedo	0.82	Hard	1
Quercus suber	0.83	Hard	1
Quercus ilex	0.9	Hard	1
Quercus pyrenaica	0.97	Hard	2
Olea europaea	0.99	Hard	3

Table S6 – Selected uncorrelated forest (F) and climatic (C) variables included in Maxent Model. Forest variables are grouped based on their mechanistic link to bat ecology: roosting availability (Roost), spatial heterogeneity (Het), forest composition (Comp), spatial clutter (Clutt). Type of variable: quantitative (Q) or categorical (C), variable unit and variable description.

Var. name	Variable group	Forest group	Type	Unit	Description
Tree DBH	F	Roost	Q	mm	Average Diameter at Breast Height of the trees.
Tree H	F	Roost	Q	m	Average height of trees.
Dead den	F	Roost	Q	%	Density of dead trees.
> 425 DBH	F	Roost	Q	trees/ha	Density of trees with DBH > 425 mm.
SoftW >425	F	Roost	Q	trees/ha	Density of softwood tree species > 425 mm DBH (Table S4).
Dev stage	F	Roost	C	-	Development stage of main tree species.
Wood density	F	Roost	Q	g/cm3	Weighted average wood density of trees.
Tree DBH ra	F	Het	Q	NA	DBH range. Thicker - thinner tree.
Tree H ra	F	Het	Q	m	Tree height range. Highest - shortest tree.
Forest type	F	Het	C		Morpho-structural type.
% plantations	F	Het	Q	NA	Proportion of plots with plantations (calculated when interpolating).
Sp1 DBH struct	F	Het	C	-	Structural heterogeneity category (regular to irregular) of the main tree species.
Sp comp	F	Comp	C	-	Type of tree specific composition.
Underst R	F	Comp	Q	n. sp	Richness of shrub species.
Broadleaved	F	Comp	Q	trees/ha	Density of broadleaved tree species.
Tree R	F	Comp	Q	n. sp	Tree species richness in plot of 25m.
Tree sp	F	Comp	C	-	Main tree species. Names shown in Fig. S10.
Sp1 D	F	Comp	Q	%	Dominance of tree Sp1 (rel. tree density).
Spatial distr	F	Het	C		Spatial distribution.
Tree C	F	Clutt	Q	%	Tree cover, estimated in percent.
Total C	F	Clutt	Q	%	Total vegetation cover, estimated in percent.
Underst C	F	Clutt	Q	% cover	Understory cover. Sum of cover of each shrub species.
Underst H	F	Clutt	Q	M	Average height of understory.
Trees/ha	F	Clutt	Q	trees/ha	Tree density (>75 DBH) in the plot.
Proportion of forest	-	-	Q	%	Proportion of 100m forest cells at 2.5km cells
Elevation	C	-	Q	m	
Annual Precipitation	C	-	Q	mm	BIO12 Bioclim variable
Temp Seasonality	C	-	Q	ºC	BIO4 Bioclim variable
Max Temp of Warm Month	C	-	Q	ºC	BIO5 Bioclim variable

Table S7- Unclustering and interpolation parameters used for each bat species. Dist uncl or: distance used to uncluster bat occurrence records. Min forest plots: minimum number of forest plots used to characterise each occurrence record. Max forest plots: maximum number of forest plots used to characterise each occurrence record. Dist max: maximum distance from forest plot to occurrence record used to select forest plots to characterise occurrence records. Dist rp: Distance used to uncluster random points. Dist from OR: Distance from occurrence records used to remove forest plots available for background selection.

**Bat species**	**Dist uncl or (km)**	**Min forest plots**	**Max forest plots**	**Dist max (km)**	**Dist uncl rp (km)**	**Dist from or (km)**
B. barbastellus	1	4	8	5	5	5
M. bechsteinii	0.5	2	5	3	4	4
M. emarginatus	0.5	2	5	3	4	4
M. escalerai	0.5	2	5	3	4	4
N. lasiopterus	2	8	16	7	5	5
N. leisleri	2	8	16	7	5	5
P. austriacus	0.5	2	5	3	4	4
R. euryale	0.5	2	5	3	4	4
R. hipposideros	0.5	2	5	3	4	4

Table S8 – Number of occurrence records for each bat species and results of their interpolation: average number of plots per occurrence record (OR) and random point (RP), and average distance from forest plots to OR and RP. Number of features in Maxent models F: (L: Linear, Q: quadratic, H: Hinge), regularisation parameter (r) and number of background points. Fig S3-S5 show the location of OR.

**Bat species**	**Occurrence records**	**Mean plots/OR**	**Mean distance/OR**	**Mean plots/RP**	**Mean distance/RP**	**F**	**r**	**Background points**
B. barbastellus	16	7.75	1.49	7.4	2.61	L	3	1537.333
M. bechsteinii	49	4.94	1.19	4.19	1.77	L	3	1639.833
M. emarginatus	98	4.64	1.5	4.16	1.79	L	3	1608.667
M. escalerai	115	4.59	1.44	4.14	1.79	L	3	1613.833
N. lasiopterus	48	15.58	2.46	14.88	3.73	LQ	3	1574
N. leisleri	48	15.75	2.32	14.86	3.77	LQ	3	1581.167
P. austriacus	123	4.62	1.42	4.16	1.78	LQ	3	1612.833
R. euryale	173	4.36	1.67	4.1	1.79	LH	3	1601.333
R. hipposideros	322	4.35	1.68	4.03	1.81	LH	3	1593.667

Table S9 – Climate variables considered prior to selection for its inclusion in models.

**Variables**	**Source**
Temperature Seasonality	www.worldclim.org
Temperature coolest month	www.worldclim.org
Temperature hottest month	www.worldclim.org
Temperature Annual Range	www.worldclim.org
Minimum April temperature	www.worldclim.org
Mean Temperature of Warmest Quarter	www.worldclim.org
Mean Temperature of Coldest Quarter	www.worldclim.org
Mean April temperature	www.worldclim.org
Mean precipitation	www.worldclim.org
Precipitation of Warmest Quarter	www.worldclim.org
Precipitation Seasonality	www.worldclim.org
Precipitation driest month	www.worldclim.org
Precipitation of Wettest month	www.worldclim.org
Precipitation of Wettest Quarter	www.worldclim.org
Elevation	www.worldclim.org
Slope	From “Elevation”
Abruptness	From “Elevation”
Distance to karst	http://arcweb.forest.usf.edu/flex/KarstRegions/
Lithology	http://ccgm.org/en/home/168-lithological-map-of-the-world-9782917310250.html
Proportion of forest	From Corine Landcover 2000 (https://land.copernicus.eu/pan-european/corine-land-cover/clc-2000?tab=download)
Human population year 2000	https://earthdata.nasa.gov/

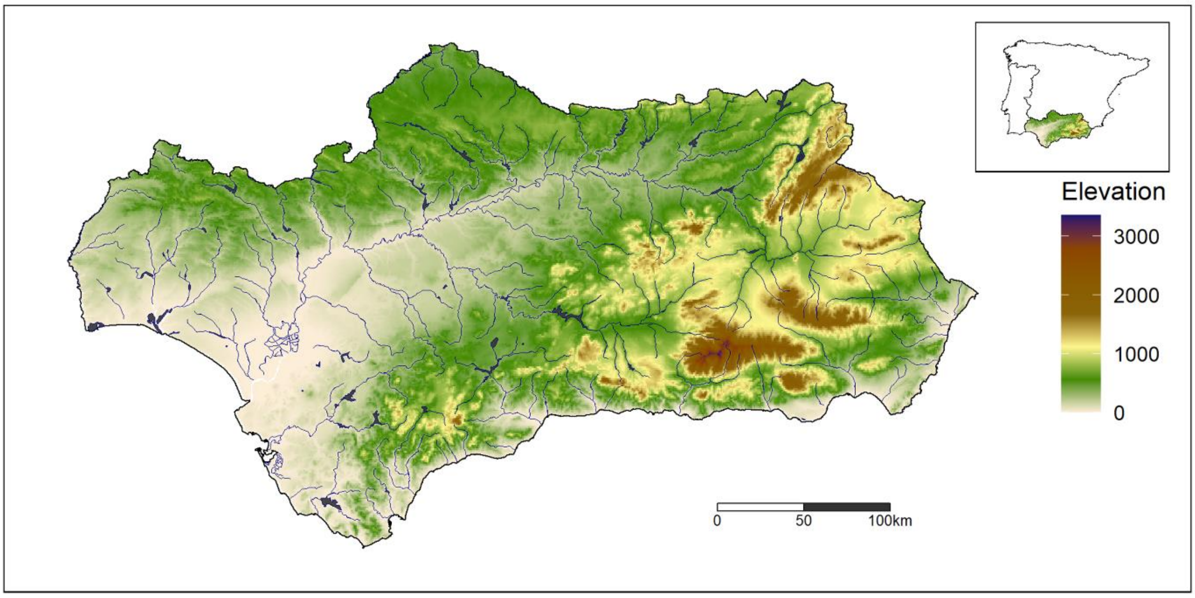

Fig. S1 – Topography of the study area in Andalusia, Spain. Generated using *ggplot2*[6] within R 4.03 (www.r-project.org/).

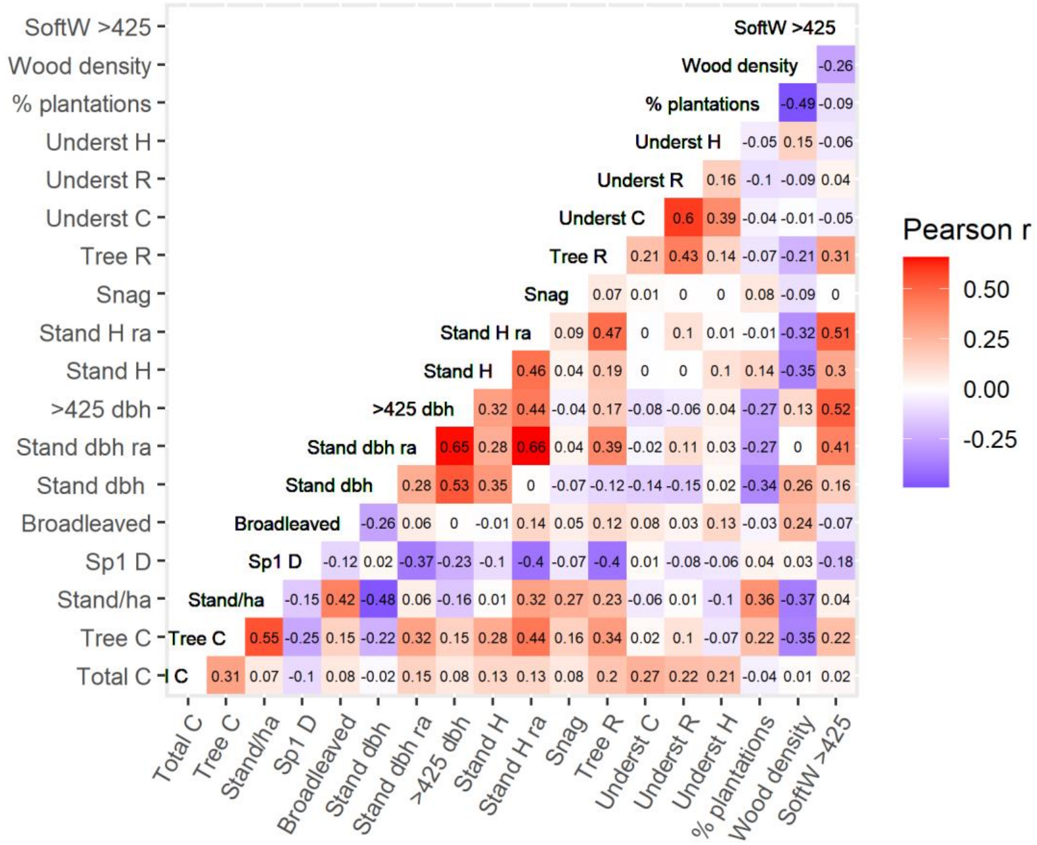

Fig. S2 – Pearson correlation coefficient *r* between all pairs of forest variables included in the study. Variable names are shown in Table S5.

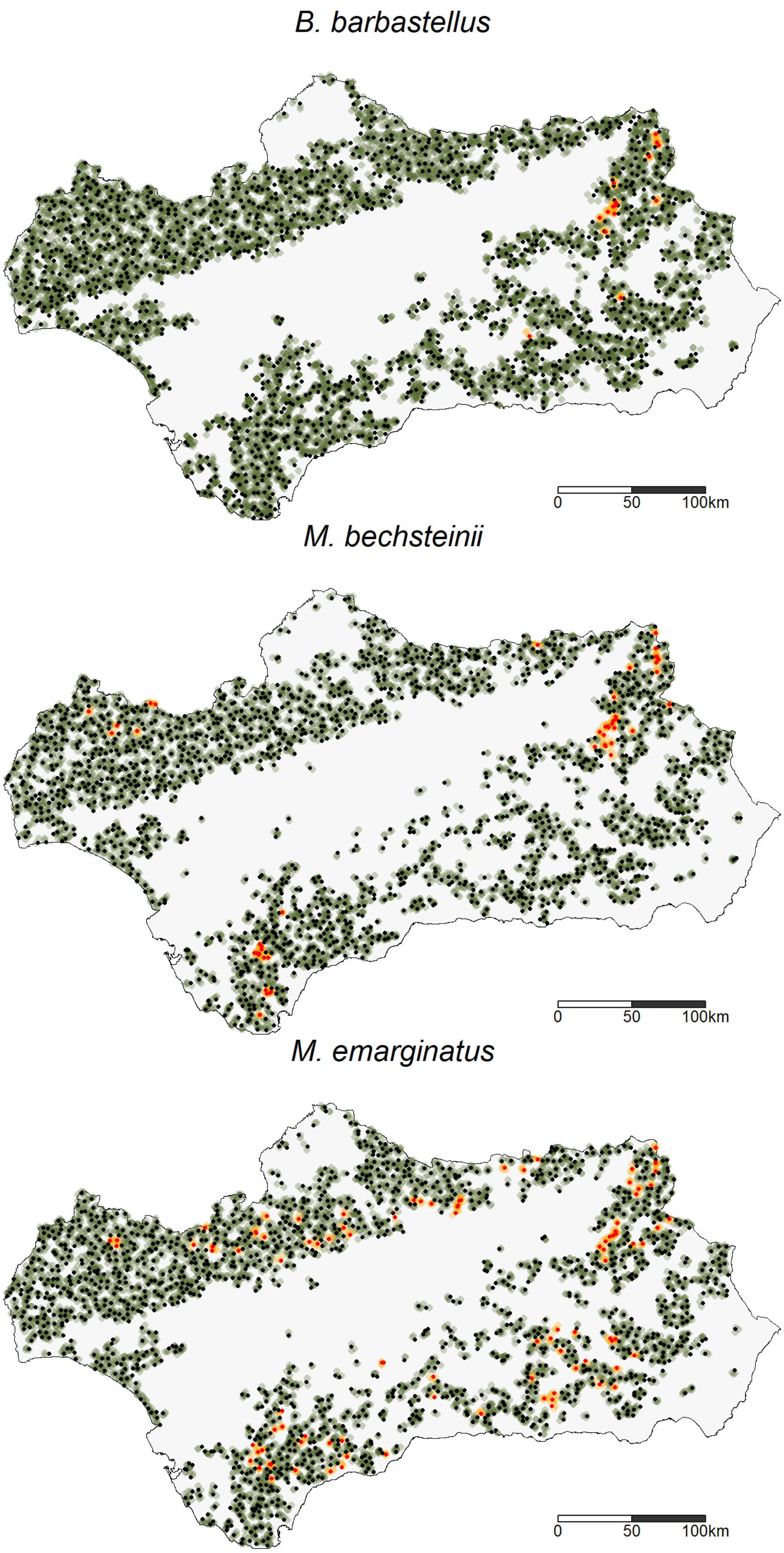

Fig. S3 – Location of occurrence records (red dots) and generated background random points (black dots) for each species together with the location of forest plots used in the interpolation on both occurrence records (orange circles) and background random points (green circles). *B. barbastellus*, *M. bechsteinii* and *M. emarginatus*. Generated using *ggplot2*[6] within R 4.03 (www.r-project.org/).

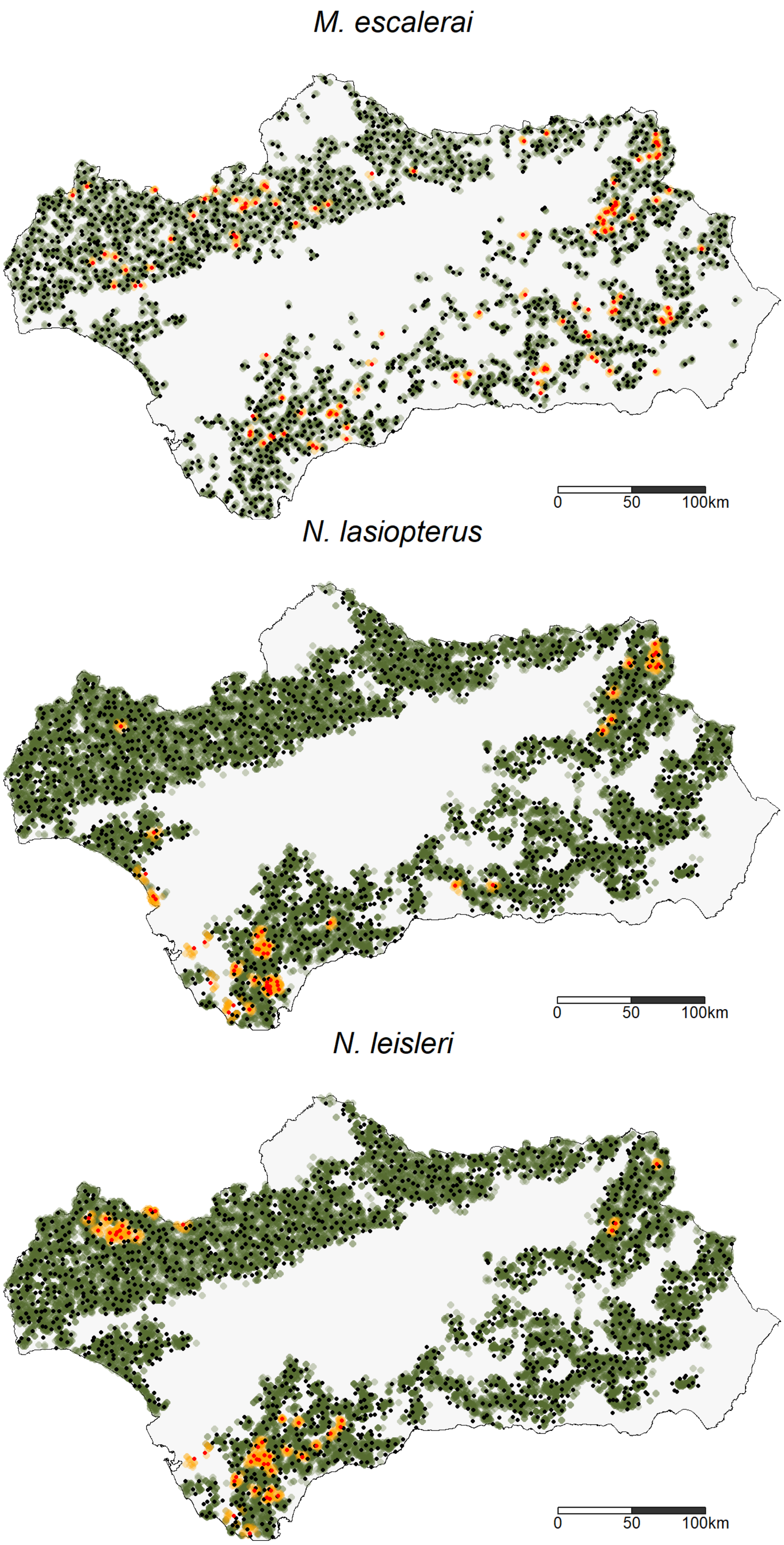

Fig. S4 – Location of occurrence records (red dots) and generated background random points (black dots) for each species together with the location of forest plots used in the interpolation on both occurrence records (orange circles) and background random points (green circles). *M. escalerai*, *N. lasiopterus* and *N. leisleri*. Generated using *ggplot2*[6] within R 4.03 (www.r-project.org/).

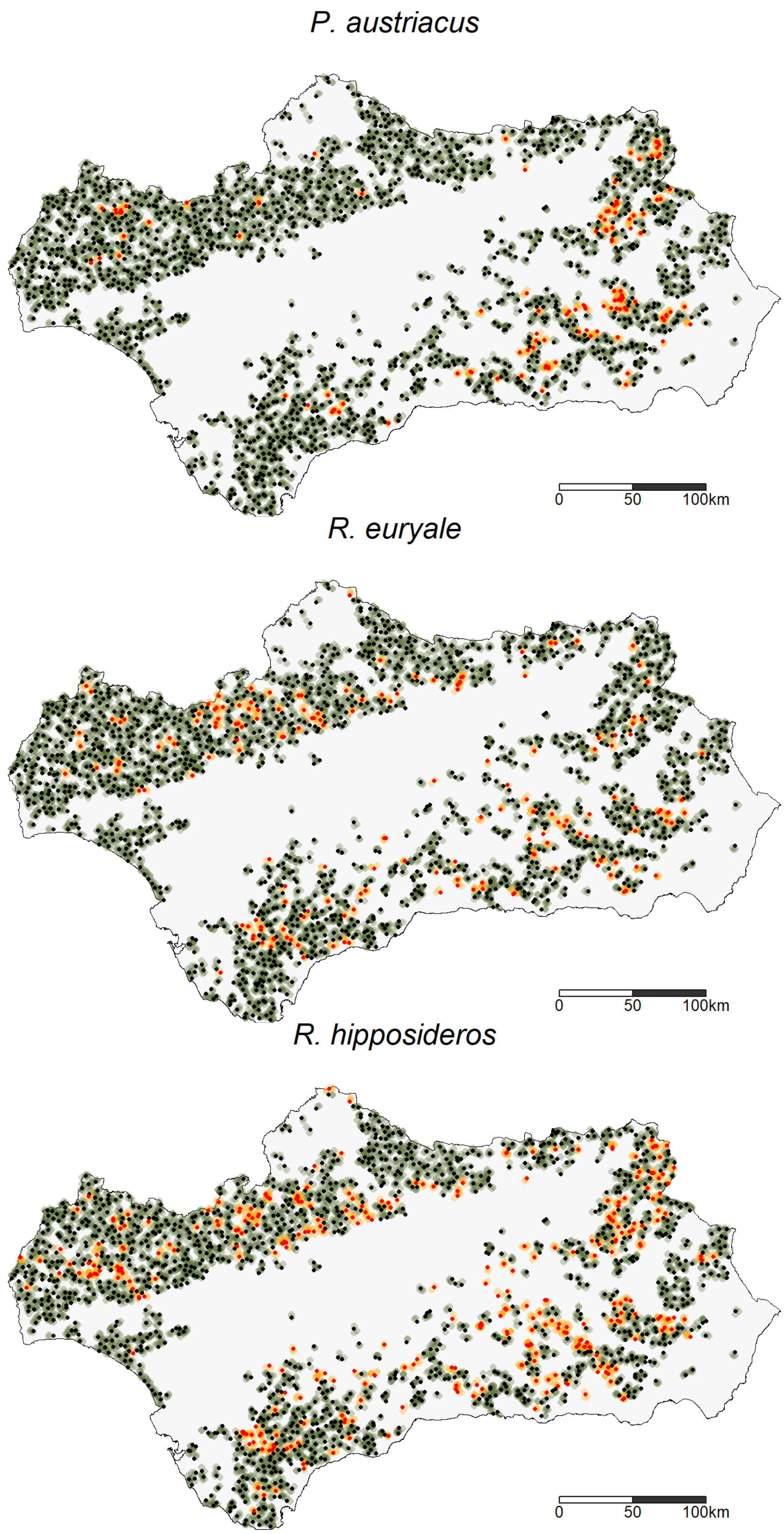

Fig. S5 – Location of occurrence records (red dots) and generated background random points (black dots) for each species together with the location of forest plots used in the interpolation on both occurrence records (orange circles) and background random points (green circles). *P. austriacus, R. euryale* and *R. hipposideros.* Generated using *ggplot2*[6] within R 4.03 (www.r-project.org/).

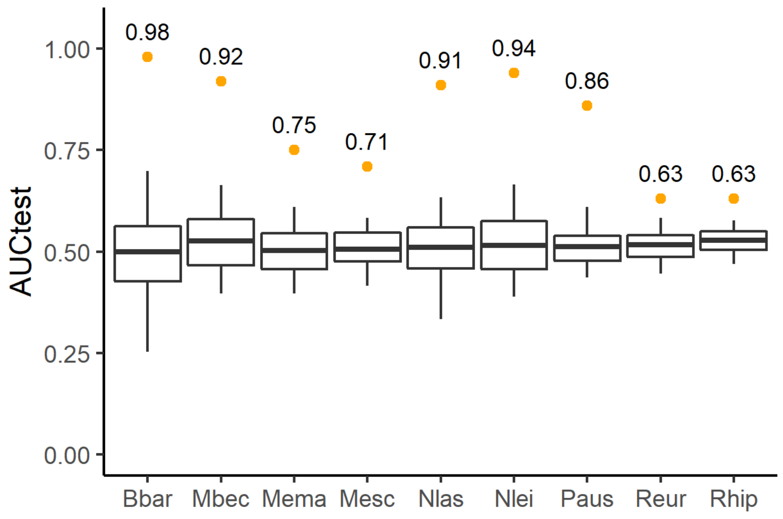

Fig. S6 – $AUC_{crossvalidation}$ values of Maxent models for each bat species (orange dots) compared to null distributions of $AUC_{crossvalidation}$ values generated by running 100 Maxent models using random presence data. Boxes represent the first and third quartiles of $AUC_{crossvalidation}$ values. Whiskers show 0.95 CI of null distribution. All cases have better fit than random (> CI 0.95). Bat species abbreviations are shown in Table 1.

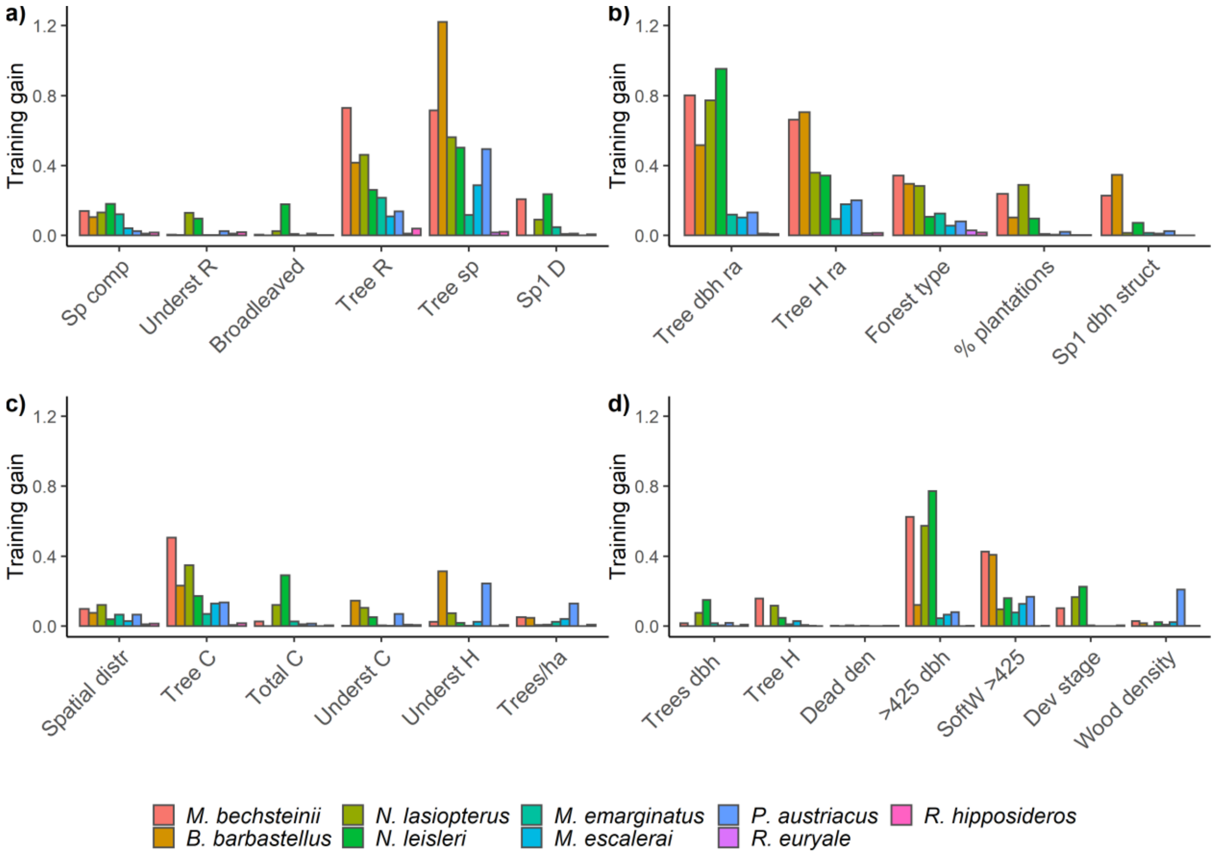

Fig. S7 – Contribution of forest variables to the models of the nine bat species based on training gain. Variables are separated into four mechanistic groups a) composition b) structural heterogeneity, c) cluttering and d) roost availability. Description of forest variables is shown in Table S5.

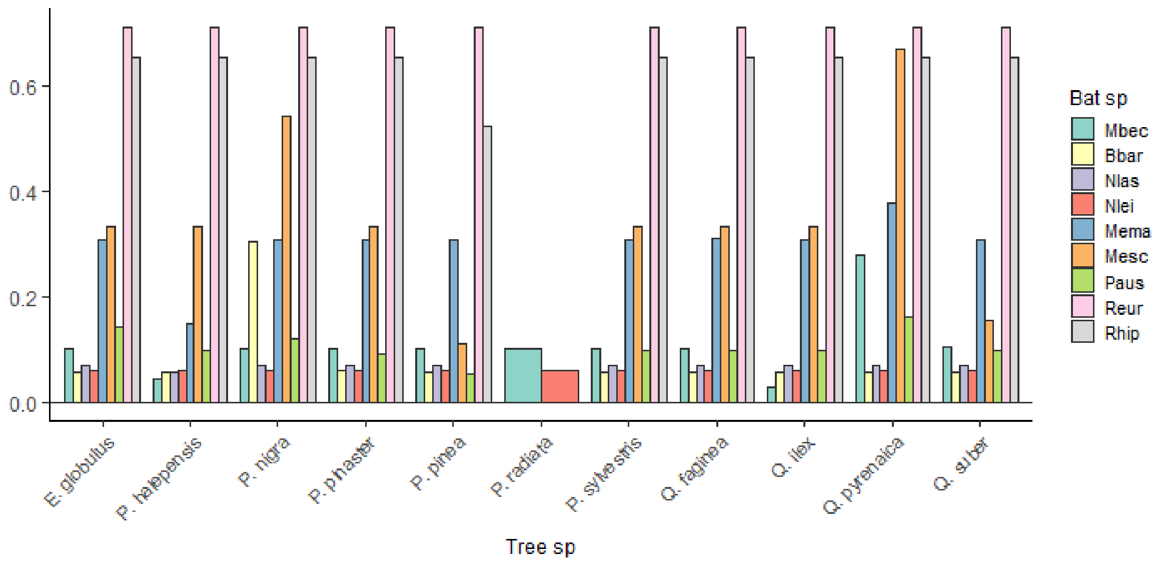

Fig. S8 – Probability of presence of each of the bat species depending on tree species Most ten common tree species are shown. Rare species in the region (dominant <1.5 % plots) are grouped as "others" and are not shown. Abbreviations of bat species names are given in Table 1.

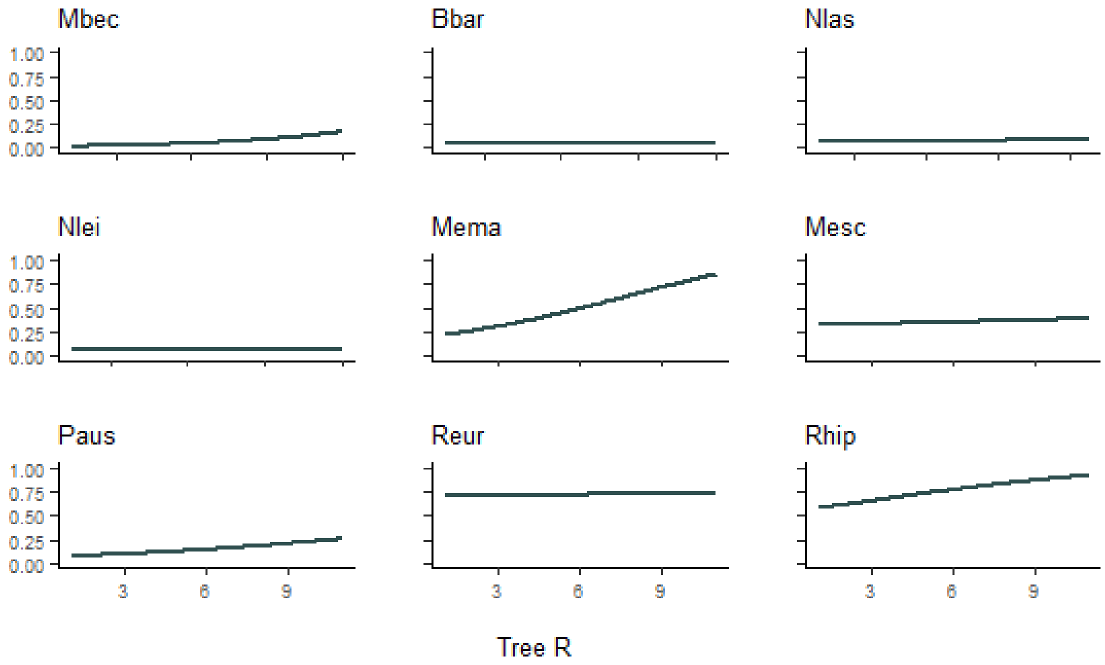

Fig. S9 – Probability of presence of each of the bat species depending on the richness of tree species. Abbreviations of species names are given in Table 1.

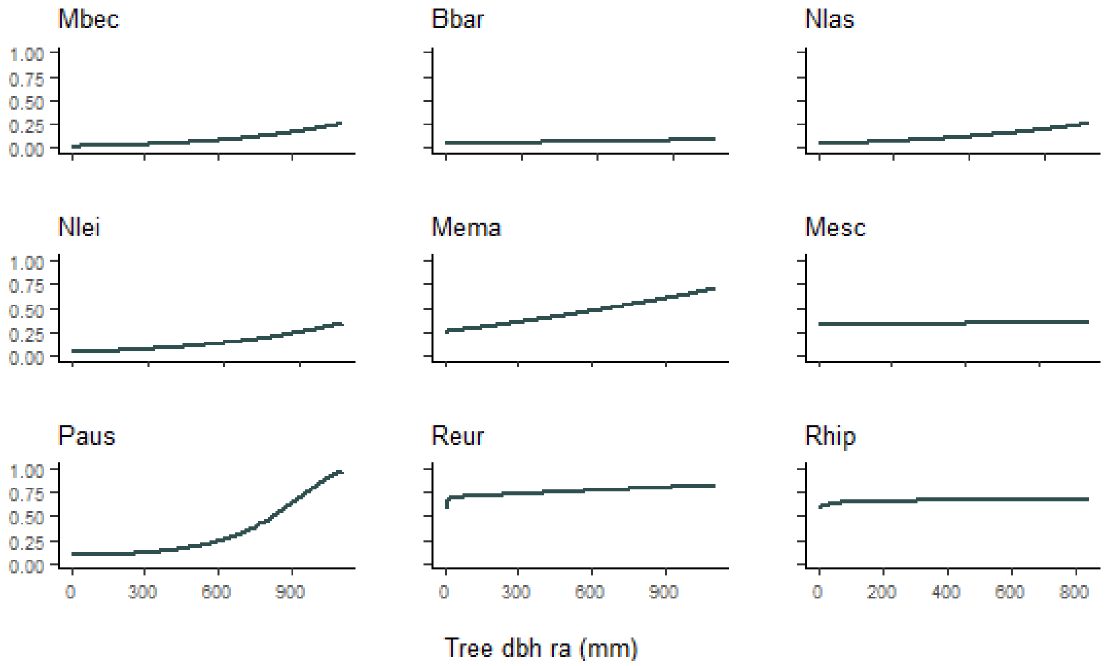

Fig. S10 – Probability of presence of each of the bat species depending on range of tree DBH. Abbreviations of species names are given in Table 1.

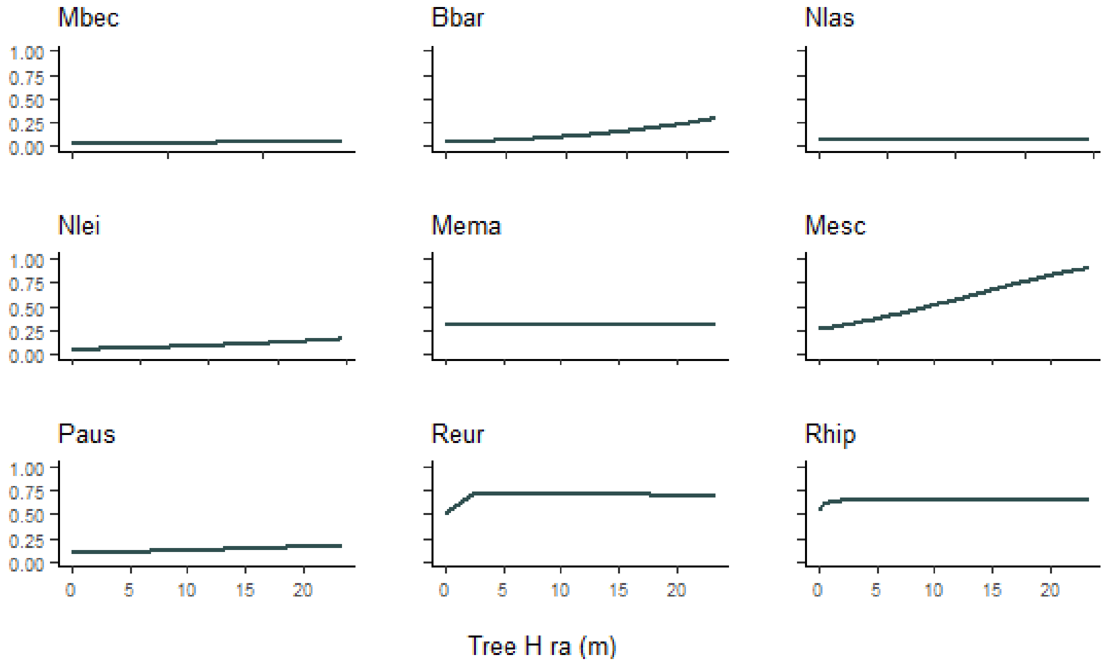

Fig. S11 – Probability of presence of each of the bat species depending on average tree height. Abbreviations of species names are given in Table 1.

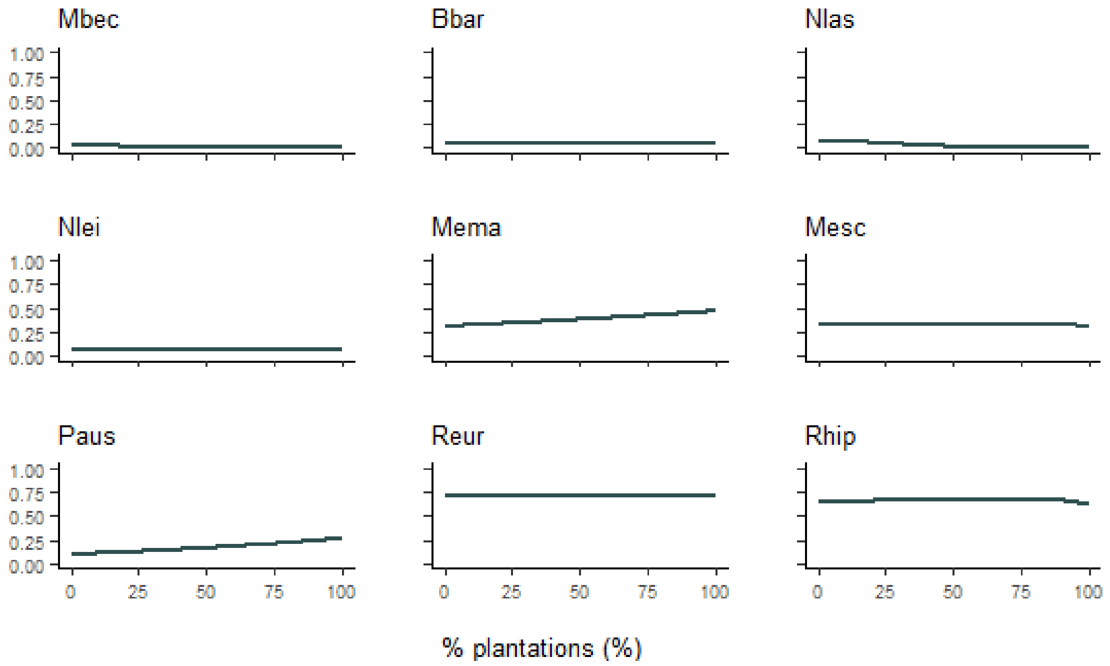

Fig. S12 – Probability of presence of each of the bat species depending on % of plantations. Abbreviations of species names are given in Table 1.

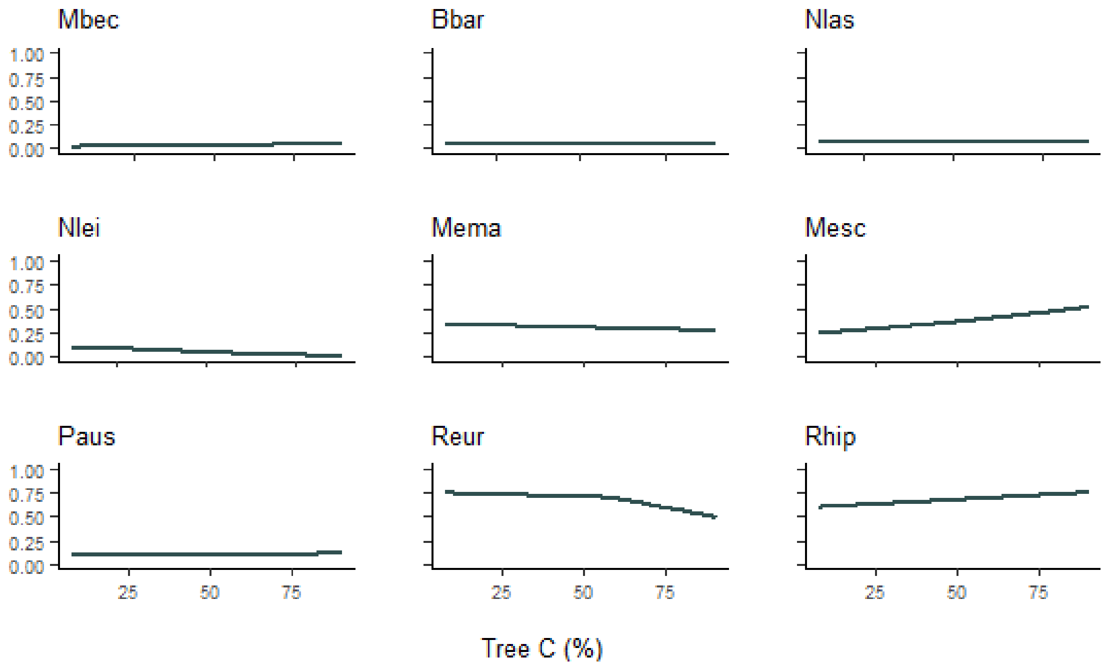

Fig. S13 – Probability of presence of each of the bat species depending on tree cover. Abbreviations of species names are given in Table 1.

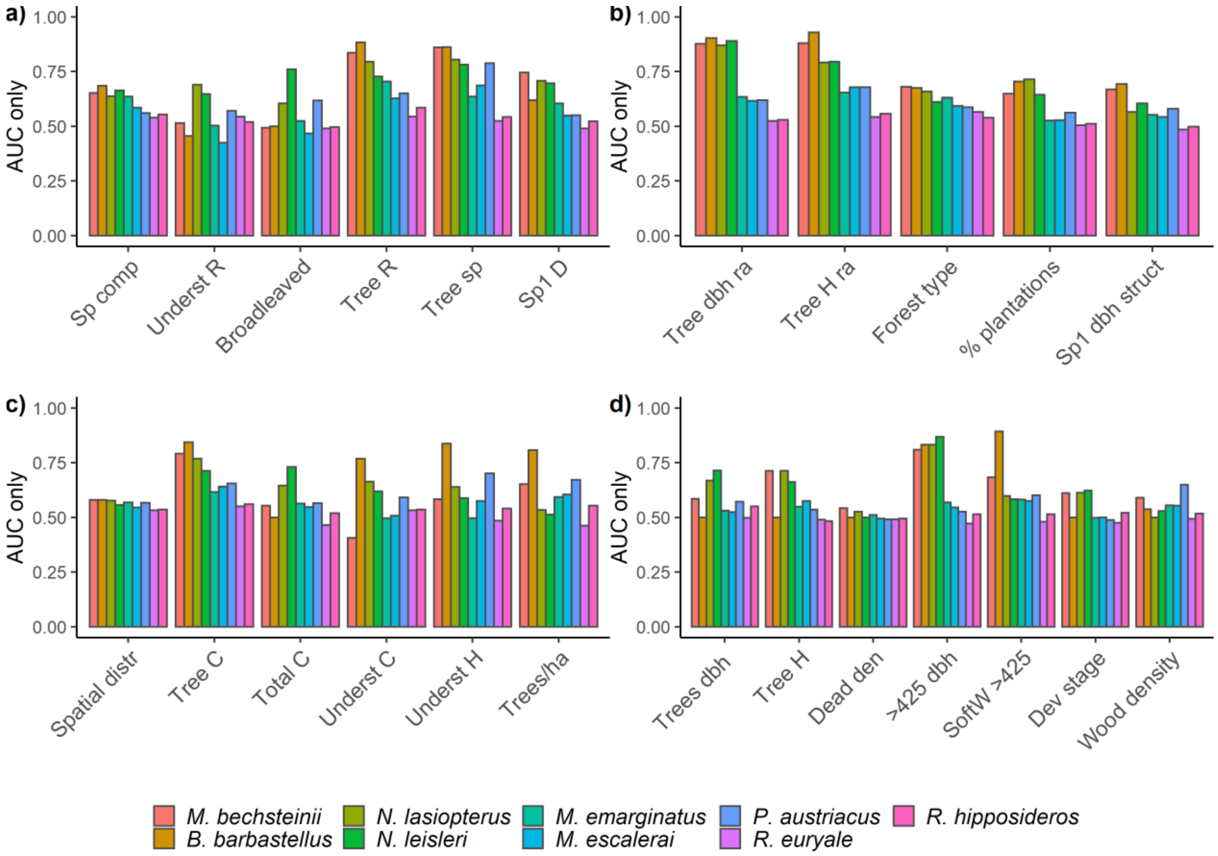

Fig. S14 – Individual AUC values for variables in Maxent models. Forest variables are separated into the following four mechanistic groups: a) composition, b) structural heterogeneity, c) cluttering and d) roost availability. Description of forest variables is shown in Table S5.

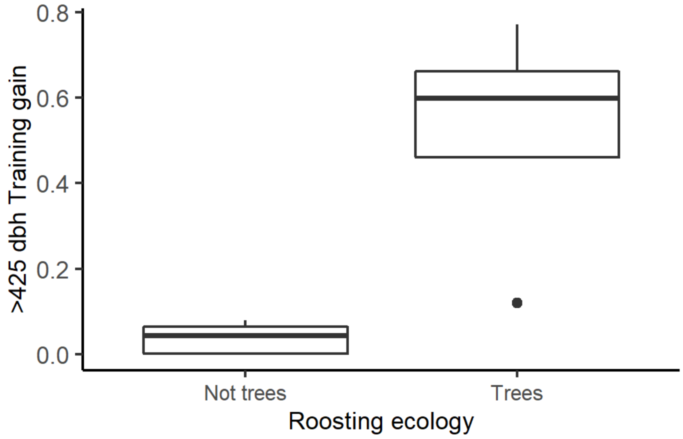

Fig. S15 – The contribution in Maxent models (training gain) of the forest variables density of trees larger than 425 mm DBH and development stage for tree roosting species versus non-tree roosting species.

References for Supporting information

1. Vayreda Duran, J., Ibàñez Martí, J. J. & Gracia, C. A. El Inventario Ecológico y Forestal de Catalunya y su consulta mediante la aplicación MiraBosc 'On-line'. *Cuad. la Soc. Española Ciencias For.* **19**, 217–227 (2005).

2. Vignote Peña, S. *Principales maderas de frondosas de España. Características, tecnología y aplicaciones*. http://oa.upm.es/30638/ (2014) doi:10.13140/2.1.3380.1606.

3. Meier, E. The wood database. https://www.wood-database.com.

4. Tejedor, C. Basic density selection for Eucalyptus globulus in northern Spain. Within-tree and between-tree variation. Carlos. *Eucalyptus a Chang. world. Proc. an IUFRO Conf.* 27–29 (2004).

5. Oramas, C. P. *Estudio de las maderas de coniferas españolas y de la zona norte de Marruecos*. (Ministerio de Agricultura. Instituto Forestal de Investigaciones y Experiencias, 1964).

6. Wickham, H. *et al.* ggplot2: Create Elegant Data Visualisations Using the Grammar of Graphics. (2020).
